# Toll-Like Receptors and Human Disease: Lessons from Single Nucleotide Polymorphisms

**DOI:** 10.2174/138920212803759712

**Published:** 2012-12

**Authors:** Yi-Tzu Lin, Amanda Verma, Conrad P Hodgkinson

**Affiliations:** Department of Medicine, Duke University Medical Center & Mandel Center for Hypertension and Atherosclerosis Research, Durham, NC 27710, USA

**Keywords:** Atherosclerosis, Genetic association studies, Inflammation, Innate immunity, Single nucleotide polymorphisms, Toll-like receptors.

## Abstract

Toll-like receptors (TLRs), a large group of proteins which recognize various pathogen-associated molecular patterns, are critical for the normal function of the innate immune system. Following their discovery many single nucleotide polymorphisms within TLRs and components of their signaling machinery have been discovered and subsequently implicated in a wide range of human diseases including atherosclerosis, sepsis, asthma, and immunodeficiency. This review discusses the effect of genetic variation on TLR function and how they may precipitate disease.

## INTRODUCTION

The immune system is comprised of two parts, the innate and adaptive systems. The innate immune system is the first line of defense against invading organisms and is highly conserved across species. Pathogens are identified on the basis of their molecular structures by a limited repertoire of so-called pattern recognition receptors (PRRs). One major family of PRRs are the Toll-like receptors (TLRs), a group of type I membrane glycoproteins evolutionarily conserved from *Caenorhadbitis elegans* to humans [1, 2]. The founding member of the family, Toll, was identified as a protein essential for embryonic dorsoventral polarity and the antifungal response of *Drosophila* [3]. TLRs are expressed on various immune cells including monocytes, dendritic cells, B cells, as well as endothelial cells [4]. Currently 12 TLRs have been characterized in mammals and they can be further divided into two groups based on their localization. TLR1, TLR2, TLR4, TLR5, TLR6, and TLR11 are found within the cell membrane and recognize components of the microbial cell wall. TLR3, TLR7, TLR8, and TLR9 are expressed internally in compartments such as the endoplasmic reticulum, endosomes and lysosomes where they bind to nucleic acids of microbial or viral origin. One of the common features of TLRs is a varying number of leucine-rich repeats (LRRs) in their extracellular domain. These LRR domains form “horseshoe-like” structures which are probably required for ligand-binding [5, 6]. The other common TLR feature is a cytoplasmic domain similar to that of the interleukin-1 receptor (termed the Toll/IL-1 R or TIR domain) [1]. This TIR domain recruits TIR-containing adaptor proteins including myeloid differentiation response gene 88 (MyD88), TRIF, TRAM, and TIRAP. These adaptors carry the signal from the receptor and are necessary for the expression of pro-inflammatory cytokines and type-I interferons [1, 4]. With the exception of TLR3, MyD88 is common to all TLR signaling pathways and induces pro-inflammatory cytokine expression through MAPK and the transcription factors NFκB, AP1, and Elk1. Mice lacking MyD88 do not activate MAPK and pro-inflammatory transcription factors in response to TLR2, 5, 7, 8, and 9 specific ligands [7-12]. TLR3 and TLR4 induce type-I interferon expression through a MyD88 independent pathway involving the TIR-domain-containing adaptor protein-inducing IFN-βprotein TRIF [13-16]. The adaptors TRAM and TIRAP are important for specificity [4, 17]; TRAM participates in the TRIF pathway of TLR4, but not that of TLR3, and TIRAP is involved in MyD88 dependent signaling for some TLRs (TLR1, TLR2, TLR4, and TLR6) but not others (TLR5 and TLR9).

One way of classifying bacteria is on the basis of differential staining of their cell walls namely Gram-negative and Gram-positive. Several components of the bacterial cell wall are TLR ligands the most potent of which is lipopolysaccharide (LPS). The process by which Gram-negative bacterial LPS activates TLR4 is well known. The first step involves LPS binding to the lipopolysaccharide-binding protein LBP. CD14, a lipid-binding protein, attaches to LPS-LBP complexes and delivers the LPS to MD-2, which in turn activates TLR4 by promoting oligomerization of the receptor [18-20]. Once LPS is bound to CD14/MD-2/TLR4 the complex acquires the signaling adaptor molecules MyD88 and TRIF. TLR2 is involved in the response to Gram-positive bacteria, does not require MD-2, and activates only the MyD88 dependent pathway [4].

## SINGLE NUCLEOTIDE SNPs

Genetic SNPs such as single nucleotide SNPs (SNPs) are common SNPs found within a population [21-28]. In the field of association genetics researchers attempt to find those SNPs which correlate with disease susceptibility. There are two types of coding region SNPs, synonymous and non-synonymous. A coding region SNP is called synonymous when the substitution produces no change in the amino acid. When a SNP results in the alteration of the encoded amino acid this is termed non-synonymous. A missense mutation changes the protein by causing a codon change. A nonsense mutation results in misplaced termination. SNPs outside the coding region can potentially affect transcription factor binding and mRNA splicing/stability, which can alter expression levels of the protein [29]. 

SNPs can be presented in several ways and as there is currently no consensus this can lead to confusion. Two examples follow which describe how SNPs are presented in this review. An example of a coding region SNP is Asp299Gly, Asp is the wild-type amino acid, 299 is the position of the altered amino acid within the protein, and Gly is the “new” amino acid arising from the SNP. For SNPs outside the coding region, the nucleotide 5’ of the ATG-translation initiation codon is -1, the previous -2, etc. For example, -159C/T is located 159bp upstream of the ATG-translation initiation codon, the wild-type base is C, and the altered base arising from the SNP is T. In the literature SNPs are often referred to in short-hard as polymorphisms or variants. 

In this review we focus on those SNPs that have been described in TLRs and their accessory proteins. A table is provided listing the SNPs for each TLR and the diseases these SNPs are associated with (Table **1**).

## TLR4 Asp299Gly AND Thr399Ile SNPs

Arbour *et al.* were the first to describe that the two common co-segregating missense mutations Asp299Gly and Thr399Ile which affected TLR4 function finding that both SNPs were associated with a blunted response to inhaled LPS in humans. Furthermore, the authors found that the TLR4 wild-type allele rescued the LPS hyporesponsive phenotype in primary airway epithelial cells and alveolar macrophages isolated from individuals carrying the TLR4 mutations [30]. 

Following the work of Arbour *et al.* [30] several research groups attempted to identify whether there was an association between these SNPs and the risk of infection. However these findings have been mixed. In an Intensive Care Unit population at risk for sepsis the TLR4 SNPs were present in a higher frequency when compared to a control population. Furthermore, there was a significantly higher incidence of gram-negative infection among patients with the mutations [31]. Similarly, infection by Gram-negative bacteria is a known risk factor for premature birth. In a Finnish population the frequencies of 299Gly allele in premature neonates were higher than term neonates [32]. In contrast, in a study following patients after major visceral surgery the presence of the Asp299Gly mutation did not correlate with the development of sepsis [33]; however the authors of this study also found in a mouse model of polymicrobial septic peritonitis a lack of TLR4 had no influence on systemic cytokine response nor the development of organ injury [33]. Likewise two studies failed to find any association between Asp299 Gly and meningococcal disease [34, 35]. Outside the bacterial milieu infants harboring the Asp299Gly and Thr399Ile mutations were found to be more susceptible to respiratory syncytial virus (RSV) [36, 37], a proposed TLR4 ligand [38]. 

A considerable body of evidence has subsequently linked the TLR4 Asp299Gly and Thr399Ile SNPs with atherosclerosis. The disease itself is believed to be dependent upon the innate immune response with TLRs being critically involved [39]. Cells of the atherosclerotic lesion express a large repertoire of TLRs [40]; where they have been proposed to have many diverse roles. For example macrophage-borne TLRs are important in converting the macrophages into foam cells; a chief component in plaque formation [41-43]. Various mouse models have also identified a role for TLRs in atherosclerosis. Acute infections arising from *Chlamydia pneumoniae*, *Helicobacter pylori*, *Porphyromonas gingivalis*, *Cytomegalovirus*, *Epstein-Barr* virus, *Herpes simplex virus*, and *HIV* have been associated with atherosclerosis [44-47]. Infection with *Chlamydia pneumoniae* promotes atherosclerotic lesion development via the formation of foam cells in mice; knockout of TLR2, TLR4, MyD88, TRIF, or IRF3 all reduced this process [41]. Deletion of MyD88, but not that of CD14, reduced early lesion development in hyperlipidemic mice by decreasing macrophage recruitment into the artery [48] as well as switching the differentiation of T cells from T_H_1 to T_H_2, which is considered to be anti-atherogenic [49, 50]. Similarly the progression of atherosclerosis was reduced in both ApoE deficient mice lacking TLR4 [51] and LDLr-/- animals without TLR2 [52, 53]. Administration of the TLR4 ligand LPS into ApoE-/- mice or hypercholesterolemic rabbits promoted atherosclerosis [54, 55] and intraperitoneal administration of a synthetic TLR2/TLR1 agonist, Pam3 CSK4, increased atherosclerosis in LDLr-/- mice [53]. Interestingly the study by Mullick *et al.* indicated that an endogenous TLR ligand was responsible for disease progression [53]. The most contentious issue regarding the putative role of infection in the development of atherosclerosis has been studies on the effect of antibiotic therapy. On the basis of the findings described above one would expect antibiotics to reduce atherosclerosis. However, several studies investigating this hypothesis have failed to show any positive effect deriving from antibiotic therapy [56-59].

The study of Kiechl *et al.* assessing the progression of carotid atherosclerosis by duplex ultrasonography found that subjects with the Asp299Gly TLR4 SNP had a lower risk of carotid atherosclerosis, smaller carotid intima-media thickness, as well as reduced levels of pro-inflammatory cytokines and acute phase reactants when compared to the wild-type control group [60]. This finding was supported by two further studies; both Ameziane *et al.* and Balisteri *et al.* identified that the Asp299Gly SNP was associated with a decreased risk of acute coronary events independently of known coronary risk factors [61, 62]. However, in other studies no association of Asp299Gly SNP with coronary artery stenosis [63] or with cerebral ischemia [64, 65] was observed. Finally, to confuse matters further, Edfeldt *et al.* found that in male myocardial infarction (MI) cases carriers of the Asp299Gly SNP were more frequent than in the male control cases [66]. Why these studies have delivered such contrasting results is unknown but possibilities have been put proposed [67]. The lack of reproducibility has been described as a potential false negative arising from phenotype differences between studies, regional differences in the study populations, and/or under-powered sample sizes [67]. 

Statins are a group of drugs that are very effective for the treatment of cardiovascular disease [68, 69]. While these drugs lower cholesterol levels by inhibiting HMG-CoA reductase, statins also have beneficial effects independent of their ability to lower lipid levels [70, 71]. The efficacy of statins has been shown to be affected by the presence of the TLR4 Asp299Gly SNP. Two clinical studies have shown that patients with the Asp299Gly SNP derive a much greater benefit from statin therapy when compared to individuals without this SNP [72, 73]. Individuals lacking the Asp299Gly SNP only slightly benefited from pravastatin (11.5% treatment group versus 18% no treatment) with respect to the risk of a coronary event within two years of the start of treatment whereas pravastatin elicited a dramatic reduction in the likelihood of a cardiovascular event from 30% to 2% in patients with the Asp299Gly SNP [72]. The synergistic effect between statins and the Asp299Gly TLR4 SNP has been ascribed to inhibition of LPS stimulation of NFκB, IL-6, and TNFα via the HMG-CoA pathway and involves Rho [74]. The ability of statins to inhibit LPS induced cytokine expression is controversial. Whereas some studies have shown inhibition [74-76] others have found no effect [77, 78]. This is perhaps due to the multiplicity of cell types, each with different LPS receptors, used in these studies.

Fibrinogen is associated with coronary heart disease, potentially as a causal factor, and promotes the production of inflammatory proteins [79, 80]. In the C3/HeJ mouse, which is null for TLR4, the ability of fibrinogen to induce inflammatory protein synthesis is compromised suggesting that fibrinogen acts in-part through TLR4 [81]. In an *in-vitro* model fibrinogen was found to activate TLR4, curiously and in marked contrast to LPS, TLR4 receptors with the Asp299Gly and/or Thr399Ile SNPs were more responsive to fibrinogen activation [82]. This intriguing finding may help explain the contradictory studies regarding TLR4 SNPs and their association with atherosclerosis. Individuals with differing levels of infection and fibrinogen would have different activity levels arising from the TLR4 receptor. This possibility is further strengthened by other putative endogenous TLR4 ligands. For instance in diabetics hyperglycemia promotes atherosclerosis via the accumulation of advanced glycation endproducts (AGEs). AGE modification of low-density lipoprotein (LDL) renders the LDL a TLR4 ligand. Neither Asp299Gly nor Thr399Ile SNP had any effect on the ability of AGE modified LDL to activate TLR4 [83].

The TLR4 SNPs Asp299Gly and Thr399Ile have also been associated with diseases other than atherosclerosis. Crohn’s disease has received a large amount of interest with respect to the TLR4 Asp299Gly and Thr399Ile SNPs. This is in part because the disease is believed to be dependent upon defects in the innate immune response [84]. In two independent cohorts the TLR4 Asp299Gly SNP was found to be significantly higher in patients with Crohn’s disease [85]. Replication studies have been mixed. The Asp299Gly SNP has been shown to be more prevalent in Crohn’s patients drawn from German [86], Dutch [87], and Greek [88] populations. No association between the Asp299Gly and/or Thr399Ile SNPs with Crohn’s disease was found in Saudi [89], Korean [90], Han Chinese [91], Tunisian [92], German [93], Hungarian [93], New Zealand [94], and Dutch [95] populations. In the Korean study the TLR4 Asp299Gly and Thr399Ile SNPs were not observed [90]. Several meta-analyses have concluded that the Asp299Gly SNP is associated with Crohn’s disease in Caucasians [91, 94, 96, 97]. It is important that additional well-powered studies are conducted in-order to conclusively determine whether the presence of the Asp299Gly SNP is a risk factor for Crohn’s disease. 

TLR4 is critical for the airway inflammatory response [100] and agents targeting TLRs are being actively pursued as novel therapies for the treatment of airway diseases such as asthma [101]. The Asp299Gly and Thr399Ile SNPs have been found to be associated with inhaled LPS hypo-responsiveness [30]. In children of European farmers Asp 299Gly was not associated with asthma [102], a finding consistent with other studies [103, 104]. Similarly, in a study using UK Caucasians no association was observed between Asp299Gly and asthma. However the atopy severity score, based on positive skin-prick tests and specific IgE, were higher in individuals carrying the Asp299Gly SNP [105]. In a Swedish study the TLR4 Asp299Gly SNP reduced IL-12 and IL-10 release from peripheral blood mononuclear cells as well as being associated with a 4-fold increase in the risk of childhood asthma [106]. 

Rare TLR4 SNPs do exist in addition to the Asp299Gly and Thr399Ile SNPs [107]. These rare TLR4 SNPs were found in significant excess in a white southern United Kingdom population with meningococcal sepsis [108]. Smirnova *et al.* [107] describe these rare SNPs as being evidence for weakly purifying selection upon TLR4; furthermore the authors raise the interesting point that mutations in TLR4 rendering the receptor hyper- or hypo-sensitive would be so deleterious, with bacteria exerting a strong selection pressure and augmented pro-inflammatory signaling being so damaging as to be unlikely to be retained in the population for the long term.

## MD-2 AND CD14

MD-2 and CD14 are essential for the function of TLR4. Genetic variation exists in both proteins. Two coding SNPs within MD-2, Thr35Ala [109] and Gly56Arg [110], have been shown to significantly decrease LPS signaling. In a study of 711 Chinese subjects a MD-2 promoter SNP was found to be significant; patients carrying the -1625 G allele were more likely to experience organ dysfunction and sepsis following a major trauma [111].

One particular CD14 SNP, -159C/T (also known as - 260C/T) has been investigated for its potential role in a wide variety of diseases. An association between the -159C/T SNP and inflammatory bowel diseases (including Crohn’s) has been noted. T allele and homozygous TT genotype frequencies were elevated in Crohn’s disease patients when compared to healthy individuals [90, 112]. The SNP had no no effect on membrane-bound or soluble CD14 levels in individuals without disease. In patients with inactive Crohn’s the TT genotype was associated with a significantly lower membrane-bound level of CD14; whereas levels of the soluble form of CD14 were higher [113]. The -159C/T SNP also appears to influence therapy for inflammatory bowel disease; cumulative anti-inflammatory corticosteroid doses were higher in ulcerative colitis patients with the TT genotype when compared to those with the CT or CC genotypes. However, this effect was not observed in individuals with active Crohn’s disease [114]. Population stratification is potentially confounding the role of CD14 in inflammatory bowel disease. In a double population cohort the -159C/T SNP was found to be associated with ulcerative colitis but not with Crohn’s in the German cohort whereas the opposite was observed in the Hungarian cohort [93]. Similarly, in an Australian cohort no association was observed between the - 159C/T SNP and Crohn’s or ulcerative colitis [115]. It is also possible that SNP(s) in linkage with the CD14 -159C/T SNP are more relevant to the disease.

Asthma, allergic rhinitis, and atopic dermatitis are diseases arising from abnormal immunoglobulin (Ig)-E responses. TT homozygotes were found to have the lowest Ig-E levels in children who had positive skin test for aeroallergens [116] and in asthmatics [117, 118]. Similarly in a Czech population the C allele was more prevalent in individuals with positive prick tests for moulds, indicating higher Ig-E levels [119]. Despite some inconsistent reports, a meta-analysis has shown that a protective effect between the - 159C/T SNP and atopic asthma susceptibility is likely to exist [120]. CD14 may act in concert with TNFα; in children with bronchial hyperresponsiveness, a synergistic effect was observed between the CD14 -159C/T and TNFα -308G/A SNPs [121]. 

One early report identified a higher frequency of the T allele in myocardial infarct survivors compared to healthy individuals [122]. However, a slew of later studies could find no link between the CD14 -159C/T SNP and the risk of myocardial infarction [123], coronary artery disease [124], or cerebral ischemia [125]. The importance of this SNP in heart disease is still an open question; even relatively recent reports give conflicting findings [126, 127]. The TLR4 Asp 299Gly SNP and CD14 -159C/T SNP, rather than either SNP separately, was found to be associated with clinically relevant atherosclerosis [128] suggesting that the CD14 -159C/T SNP may influence heart disease only in concert with variation in other genes.

## MyD88, TRIF, TRAM, TIRAP

A study characterizing sequence variation within the MyD88, Trif, Tram, and Tirap adaptors in healthy individuals from sub-Saharan Africa, Europe, and East Asia has shown that MyD88 and TRIF have evolved under purifying selection indicating deleterious SNPs within these two genes would not survive for long. The other adaptors showed signs of adaptive evolution restricted to specific populations [129]. This may explain the few reports linking SNPs within MyD88 and TRIF with human diseases [130, 131]. In one such study, children carrying rare SNPs within MyD88 (in-frame MyD88 deletion or missense mutations Leu93Pro Arg196Cys) suffered from very severe pyogenic bacterial infections [132]. These SNPs blocked fibroblast chemokine /cytokine synthesis. The MyD88 Ser34Tyr and Arg98Cys SNPs prevent complex formation between MyD88, IRAK4, and IRAK1/2 severely reducing NF-κB activation [133]. In a study of over 6000 individuals from European, South-east Asian, and African nations heterozygosity for the Tirap Ser180Leu SNP was associated with protection from tuberculosis, malaria, bacteremia, and invasive pneumococcal disease through decreased TLR2 signalling [134]. Despite replication in other populations [135], a recent meta-analysis has concluded that the Tirap Ser180Leu SNP is unlikely to be important for susceptibility to tuberculosis [136]. The Tirap Ser180Leu SNP has also been associated with Behcet’s disease [137], sepsis [138], and Chagas cardiomyo pathy [139]. The synonymous Tirap SNP, C558T (Ala186 Ala), has also been linked to increased susceptibility to tuberculosis. Individuals with the 558TT genotype had decreased levels of interleukin-6 in whole-blood [140].

## TLR1

The non-synonymous TLR1 SNP Ile602Ser is important for the ability of the receptor to signal. This is likely to be due to an inability of the 602Ser SNP to traffic to the cell surface [141]. In a whole-blood cytokine assay, lipopeptide stimulation resulted in higher levels of IL-6 with individuals carrying the 602IleIle phenotype when compared to the 602SerSer SNP [142]. Functionally, the 602Ser SNP protects against leprosy [141] and increases susceptibility to candidemia [143]. Other TLR1 SNPs include Asn248Ser [144] and -7202A/G [145]. The Asn248Ser SNP diminishes TLR1 signalling and has been linked to leprosy. Curiously, the presence of two copies of the Asn248Ser SNP was positively associated with leprosy, whereas the heterozygous state (i.e. one copy of the SNP) was negatively associated with the disease [144]. The TLR1 -7202A/G SNP increases TLR1 cell surface expression and augments TLR1-mediated NF-κB activation. In a cohort of patients with sepsis and sepsis related acute lung injury this SNP predicted a higher likelihood of organ dysfunction and death [145]. 

## TLR2

The TLR2 Arg677Trp mutation was identified in individuals with lepromatous leprosy. Cell lysates from *Mycobacterium leprae* induced monocytes to synthesize IL12, and monocytes from patients with the Arg677Trp SNP produced a lower amount of this cytokine [146]. This finding was confirmed in transfection assays involving HEK293 cells [147].

Arginine to glutamine substitution at residue 753 influences the risk of tuberculosis [148]. This SNP also reduces the ability of the receptor to respond to ligands, as individuals heterozygous for the mutation produce less cytokines when stimulated with Borrelia lysate [149]. The mutation protects against Lyme disease [149] but carriers are more susceptible to urinary tract infections [150]. Lorenz *et al.* linked the Arg753Gln mutation with an increased risk of sepsis, and linked the finding to staphylococcal infections [151]. However, a somewhat larger study could find no link between this SNP and severe infections with *Staphylococcus aureus* however [152].

Stimulation of whole blood from two donors heterozygous for the Arg477stop SNP displayed lower secretion of pro-inflammatory cytokines when compared to control [153].

## TLR3

Of the over 100 single nucleotide SNPs within the TLR3 gene four result in amino-acid substitutions; Asn284Ile, Tyr307Asp, Leu412Phe, and Ser737Thr. None of these four SNPs were found to be linked to each other. The Leu412Phe SNP is very prevalent and found in 20% of one sampled population with values reaching as high as 50% in patients with enteroviral myocarditis/cardiomyopathy [154]. Intriguingly the Leu412Phe SNP has been identified as a prognostic marker for stage II colorectal cancer; individuals homozygous for the mutation had a significantly higher risk of death from the disease [155]. The 412Phe mutation may also protect against advanced “dry” age-related macular degeneration [156] and potentially confers resistance to HIV-1 infection [157].The Leu412Phe and Ale284Ile SNPs inhibit the receptor from signaling by preventing cell surface localization [158]. A rarer SNP, Pro554Ser, was found in two patients with herpes simplex encephalitis but absent in 1581 healthy individuals and impaired the ability of the receptor to induce NF-κB and IRF-3 in response to poly(I:C) [159]. 

## TLR5

Flagellin, the TLR5 ligand, is the main pro-inflammatory stimulus in lung epithelial cells. The common TLR5 SNP, Arg392Stop, prevents flagellin from signaling and increases susceptibility to pneumonia as caused by *Legionella pneumophila* [160]. The 392Arg allele is preferentially transmitted to systemic lupus erythematosus (SLE) affected offspring, indicating that the stop codon may provide protec tion against the disease [161]. A replication study, however, failed to find any association between the Arg 392Stop SNP and the disease [162]. In a Jewish population. the stop codon protected against Crohn’s disease [163] SNP.

## TLR6

TLR6 contains a coding SNP in the extracellular domain that converts Serine-249 to Proline. This SNP potentiates IFN-γ release following injection of the Bacillus Calmette-Guérin (BCG) vaccine [164]. The Ser249Pro SNP has been associated with a decreased risk of asthma in a nested case-control disease-association study involving African-Americans [165]. Another study in Germany found a weak association of the TLR6 Ser249Pro SNP and asthma, however this association was lost after Bonferroni correction [166]. In single studies the TLR6 Ser249Pro SNP has been linked with susceptibility to infection with aspergillosis [167] and women homozygous for the 249Ser allele were found to have thinner left ventricular posterior walls as well as possessing monocytes responding weakly to the TLR6 ligand zymosan [168].

## TLR7

The study of Schott *et al.* identified three single nucleotide SNPs in TLR7 with an allele frequency of greater than 5%, these being Gln11Leu, 1-120T / G found in intron 1, and a synonymous SNP which had did not change the amino acid at codon 801[169]. The coding SNP Gln11Leu and the intron SNP affect Hepatitis C virus (HCV) infection. The intron SNP 1-120T/G was protective against the inflammation and fibrosis arising from chronic Hepatitis C infection in males [169]. The GlnLeu11 SNP was found not to affect HCV viral load but decreased IFN-λ expression and was associated with disease induced portal lymphoid aggregates [170]. Presence of the Gln11Leu SNP has also been associated with HIV infection; virus titer was higher and progression of the disease enhanced in carriers of the SNP [171]. Studies examining the role of this coding SNP in systemic lupus erythematosus have been mixed, an association [172] was found in East Asians but none was observed in Spanish Caucasians [173].

## TLR8, TLR9, TLR10

The TLR8 SNPs Met1Val and -129G/C render individuals more susceptible to Crimean-Congo hemorrhagic fever [174] and tuberculosis [175]. No association has been found between these SNPs and coronary artery disease [176].

Over 60 publications have linked the TLR9 mutations -1486 T/C, -1237 T/C, +1174 G/A, and/or 2848 A/G to the predisposition of a wide variety of inflammatory disorders including non-Hodgkin’s lymphoma [177], cervical cancer [178], lupus nephritis [179], and cerebral malaria [180], amongst many others. Most of these studies have currently not been duplicated and consequently await verification. The -1237T/C SNP generates a functional IL-6 response element. Mononuclear cells carrying this SNP when exposed to IL6 respond by producing more TLR9 which then exacerbates the cellular response to the TLR9 ligand CpG [181]. Intriguingly, one study in Han Chinese children did not detect the -1237 T/C SNP [182]. The relatively rare TLR9 SNP Pro99Leu prevents the receptor from being activated by ligands despite ligand binding being normal indicating a biphasic model to TLR9 activation whereby the receptor first senses the ligand and then responds [183].

For TLR10, two SNPs Pro344Pro and Iso775Val have been found to be associated with asthma in a study of 47 subjects [184]. Several SNPs within the TLR10 gene have been linked to Crohn’s disease [185, 186]. 

Since the discovery of TLR11, no association studies have been published linking the receptor to a disease. However, mice lacking the TLR11 gene are highly susceptible to kidney infection by uropathogenic bacteria [187]. 

## CONCLUSIONS

In reviewing the literature it is clear that the role of TLR mediated inflammation is important for numerous human diseases. However, the association studies described herein raises several important and interesting questions. 

The selection pressure exerted by foreign agents, such as bacteria, has been so great and pervasive throughout human history that one would expect that mutations which prevented the immune system from activating would be so deleterious as to disappear from the population relatively rapidly, a conclusion put forward by Smirnova *et al.* in their study of rare TLR4 SNPs [107, 108]. How would individuals with common mutations, such as the TLR4 Asp299Gly SNP, which are believed to have a negative impact on the receptor, have survived infections in the past? Several reports have indicated that TLR SNPs confer a protective advantage upon individuals that carry them. These benefits may be sufficient to over-ride the strong negative selection pressure arising from a weakened immune system. One issue has been the conflicting nature of many of these association studies described in this review. For example the TLR4 Asp299Gly SNP has been associated with heart disease in some studies but not others. Sample populations are often small and differ in their origins; conclusions may not be able to be drawn until large studies are completed in several diverse populations. The problem of confounding results, as well as the fact that many of these association studies await replication, has prevented the use of TLR SNPs as diagnostic tools. However, it is almost certain that TLR SNPs will be important in the area of personalized medicine. For example, it has been shown in several studies that the TLR4 Asp299Gly SNP has a dramatic bearing on the efficacy of statins [72, 73]. Similarly the CD14 -159C/T SNP necessitates the need for higher corticosteroid doses in individuals suffering from inflammatory bowel disease [114]. 

Another potentially confounding issue has been highlighted by Ferwerda *et al.* [188]. Many studies with the TLR4 Asp299Gly and Thr399Ile SNPs treat each SNP separately neglecting that the SNPs are co-segregated. The TLR4 haplotypes were found have different effects on lipopolysaccharide stimulation of whole blood for example [189]. 

Explaining how these TLR SNPs affect these various diseases described above is likely to be a complex question. Taking again TLR4 as an example, lipopolysaccharide (LPS) activates the receptor. Studies on the effect of the common TLR4 mutations Asp299Gly and Thr399Ile upon LPS activation have been mixed. Reports have shown that the SNPs inhibit LPS activation [30] whereas others have seen no difference in the ability of ”wild-type” and “SNP” receptors to be stimulated by this ligand [190]. *In vitro* assays investigating TLR functions have been shown to be prone to conflicting results arising from bacterial contamination of the ligands used in the experiments [191]. Another complexity arises from endogenous ligands, which may not be as similarly affected by SNPs as their pathogen counterparts. As described above AGE-LDL and fibrinogen have both been shown to activate TLR4 [82, 83]. AGE-LDL signaling was un-affected by the Asp299Gly and Thr399Ile SNPs, indeed these SNPs curiously augmented the ability of fibrinogen to signal through the receptor. Though the validity of endogenous TLR ligands has been questioned [191] their probable existence, and their interaction with TLR SNPs, will need to be evaluated when trying to explain the findings of association studies. Finally, the majority of studies have used monocytes for their investigations into how SNPs affect TLR function. However, other cell types, such as endothelial cells, also express TLRs. It is an open question whether SNPs in the receptors borne by these cell types would be similarly affected as monocytes.

## Figures and Tables

**Table 1.** Diseases Linked to SNPs of TLRs and Their Accessory Proteins

Gene	SNP	Disease	Association Found	Association Not Found
TLR1	-7202G/A	Sepsis	[145]	
	Asn248Ser	Leprosy	[144]	
	Ile602Ser	Leprosy	[141]	
		Candidemia	[143]	
TLR2	Arg677Trp	Leprosy	[146]	
	Arg753Gln	Tuberculosis	[148]	
		Lyme disease	[149]	
		Urinary tract infection	[150]	
		Staphylococcal infection	[151]	[152]
TLR3	Asn284Ile	None		
	Tyr307Asp	None		
	Leu412Phe	Colorectal cancer	[155]	
		Macular degeneration	[156]	
		HIV infection	[157]	
	Pro554Ser	Herpes simplex encephalitis	[159]	
	Ser737Thr	None		
TLR4	Asp299Gly	Atherosclerosis	[60]	[63]
		Infection	[31]	[33]
		Crohn’s disease	[85]	[94]
		Asthma		[105]
	Thr399Ile	Atherosclerosis	[60]	[63]
		Infection	[31]	
		Crohn’s disease		[94]
TLR5	Arg392Stop	Pneumonia	[160]	
		Systemic lupus erythematosus	[161]	[162]
TLR6	Ser249Pro	Asthma	[165]	[166]
		Aspergillosis infection	[167]	
		Left ventricular wall thinning	[168]	
TLR7	Gln11Leu	Hepatitis C infection	[170]	
		HIV infection	[171]	
		Systemic lupus erythematosus	[172]	[173]
	1-120T/G	Hepatitis C infection	[169]	
TLR8	Met1Val, -129G/C	Congo hemorrhagic fever	[174]	
		Tuberculosis	[175]	
		Atherosclerosis		[176]
TLR9	Pro99Leu	None		
	-1237T/C	Non Hodgkins lymphoma	[181]	
		Cerebral malaria	[180]	
	+1174G/A	Lupus nephritis	[179]	
	+2848G/A	Cervical cancer	[178]	
TLR10	Pro344Pro	Asthma	[184]	
	Iso775Val	Asthma	[184]	
TLR11	None reported			
CD14	-159C/T	Crohn’s disease	[112]	[115]
		Asthma	[120]	
		Atherosclerosis	[122]	[124]
MD-2	-1625C/G	Organ dysfunction, sepsis	[111]	
MyD88	Leu93Pro	Infection	[132]	
	Arg196Cys	Infection	[132]	
Tirap	Ser180Leu	Tuberculosis	[134]	[136]
		Malaria	[134]	
		Bacteremia	[134]	
		Bahcet’s	[137]	
		Sepsis	[138]	
		Chagas cardiomyopathy	[139]	
	Ala186Ala	Tuberculosis	[140]	
